# Table of Meteorological Conditions in the City of Buffalo for August, 1854

**Published:** 1854-10

**Authors:** Sanford B. Hunt


					﻿ART. VI.— Table of Meteorological Conditions in the City of Buffalo for
August, 1854. By Sanford B. Hunt, M. D.
i	. Upper Station. Lower Station.
.5	«5	£	Hygrometer.	Hygrometer
. S	Q.£	,------—-------------. ,------------------'
-g o O g6 'o
e 3 *S * 2 p- V	3 gg
®	o	,s	gs	g	£	8	g	>	8
rt	«	2	'g c • 2	o	o	s	<u	«	p
Q ri Q 5	£	HQ Q H Q M
___________________ <1 __________ ________________________________________________
1	29.2 1 05	7	l.S. W.	80.	73.	811	81.	71.66	765	5
2	29.2	2	2.S. W.	78.	57.	530	79.	51.	425	11
3	29.2	1	2.S. W	75.	53.33	618	77.	53.66	503	10
4	29.2	0	2-S.S.W.	76.	59.33	614	75.	63.33	694	15
5	29.2	0	4.S. W.	77.	60.66	616	73.	63.66	763	14
6	10	2.N.W.	75.	61.	654	72.	60.33	697	9
7	29.	2	2.N.	69.	52.66	609	68.	49.33	558	13
8	29.2	2	l.W.	69.	48.	524	71.	52.33	558	4
9	29.2	3	l.S.	7.3.	52.	530	70.	53.33	620	6
10	29 2	3	l.W.	78.	54.66	503	75.	58.33	618	10
11	29.1	5	l.W.	79.	65.	656	76.	62.	650	4
12	29.1	3	3.W.	79.	65.	656	78.	64.	655	5
13	3	2.W.	82.	70.33	691	79.	62.33	607	2
14	29.2	2	3-W.	76.	59.66	614	74.	53.	530	7
15	29.3	0	3.W.	75.	56.33	563	75.	54.	530	11
16	29.2	72.	51.	530	72.	55.33	619	3
17	29.3	-6	~	73.	55.33	558	70.	53.33	620	4
18	29.3	5	5	o	72.	54.33	560	70.	53.33	620	6
19	29.3	8	8	8	77.	60.33	616	74.	60.	657	7
20	G «	«	78.	66.33	686 76.	62.	650	5
21	29.3	>	>	£	78.	64.	655	76.	64.33	694	8
22	29.3	79.	69.66	738	80.	70.66	765	9
23	29.2	71.	59.33 , 697	77.	63.	650	4
24	29.2	79.	69.66	738	78.	68-66	758	5
25	29.2	77.	65.33	696	76.	66.66	758	12
26	29.2	77.	72.33	810	72.	53.33	560	5
27	75.	63.33	694 77.	58.33	565	7
28	29.2	76.	52.66	503	77.	58.33	565	5
29	29.2	79.	62.	656	80.	63.33	618	4
30	29.2	81.	64.33	619	81.	67.	657	3
31	29.2_____________________79.	67.33	686	81.	64.33	619	2
Mean,.... 29.2________ 75.61 59.62	633_75.48	59.81	630	214~
				

